# Social Determinants of Perceived Discrimination among Black Youth: Intersection of Ethnicity and Gender

**DOI:** 10.3390/children5020024

**Published:** 2018-02-15

**Authors:** Shervin Assari, Cleopatra Howard Caldwell

**Affiliations:** 1 Department of Psychiatry, University of Michigan, Ann Arbor, MI 48104, USA; 2 Center for Research on Ethnicity, Culture and Health, School of Public Health, University of Michigan, Ann Arbor, MI 48104, USA; cleoc@umich.edu

**Keywords:** African Americans, socioeconomic status (SES), income, financial difficulty, discrimination

## Abstract

Most of the existing sociological and epidemiological literature has focused on the protective effects of high socioeconomic status (SES) on population health through reducing exposure to risk factors and increasing human and material resources that can mitigate adversities. Recent studies, however, have documented poor mental health of high SES Blacks, particularly African American males and Caribbean Black females. The literature also shows a link between perceived discrimination and poor mental health. To better understand the extra costs of upward social mobility for minority populations, this study explored ethnic by gender variations in the associations between SES indicators and perceived discrimination in an ethnically diverse national sample of Black youth. This study included 810 African American and 360 Caribbean Black youth who were sampled in the National Survey of American Life—Adolescent supplement (NSAL-A). Three SES indicators (financial hardship, family income, and income to needs ratio) were the independent variables. The dependent variable was perceived (daily) discrimination. Age was the covariate. Ethnicity and gender were the focal moderators. Linear regressions were used for data analysis in the pooled sample and also based on the intersection of ethnicity and gender. Considerable gender by ethnicity variations were found in the patterns of the associations between SES indicators and perceived discrimination. Financial hardship was a risk factor for perceived discrimination in African American males only. High family income and income to needs ratio were associated with high (but not low) perceived discrimination in African American males and Caribbean Black females. SES indicators were not associated with perceived discrimination for African American females or Caribbean Black males. When it comes to Black youth, high SES is not always protective. Whether SES reduces or increases perceived discrimination among Black youth depends on the intersection of ethnicity by gender. Additional research is needed to understand why and how high SES increases exposure and vulnerability to discrimination for some groups of Black youth.

## 1. Introduction

Most of the sociological and epidemiological literature has focused on protective effects of high socioeconomic status (SES) on population health [1,2,3]. Protective effects of SES indicators such as income on physical and mental health of populations have been shown over and over by multiple longitudinal studies including the Health and Retirement Study (HRS) [4], the Americans’ Changing Lives (ACL) Study [5,6,7,8], the Panel Study of Income Dynamics (PSID) [9], the British Cohort Study (BCS) [10] and the Survey of Health, Aging and Retirement in Europe (SHARE) [8]. Mirowsky and Ross have described the effects of SES on health as “enduring, consistent, and growing” [10]. The main mechanism by which high SES protects health is through enabling individuals to have higher access to human and material resources, avoid risk of illness, and minimize their consequences when they occur [11,12,13,14].

Although on average, high SES is protective against poor health [11,12,13,14], the health gain associated with high SES may be diminished for Blacks. In a national sample, education was associated with drinking behaviors of White but not Black adults [15]. Using the Health Retirement Survey (HRS), a study showed that among adults over age 50, high income was protective against sustained high body mass index (BMI) among White women and Black women, but not White men and Black men. High education attainment was also protective against insomnia, physical inactivity, and BMI for White men, White women and Black women but not for Black men [16]. In the ACL data, education attainment had a smaller effect on life expectancy for Blacks than for Whites [15]. Again, in the ACL data, employment showed a large boost to the life expectancy of White men but had almost no effect for Black men [17].

In addition to the diminished health gain from SES, there are even studies documenting lower mental health status of individuals with high SES Blacks [18,19,20]. These studies suggest that upward social mobility may be associated with some extra psychological and social costs for Blacks [15,16,17,18,19]. To give some examples, using the ACL data, a 25-year longitudinal study with a national representative sample, African American men with high education credentials were the only group who experienced an increase in depressive symptoms over the course of follow up; however, this phenomenon was not observed for White males, White females, or African American women [18]. In a very recent study, Wilson, Thorpe, and LaVeist used the Medical Expenditure Panel Surveys (MEPS) data and showed that very large health disparities exist between health of high SES Blacks and Whites, defined as having income of $175,000 and above. Findings revealed health disparities in 10 of the 16 health-related outcomes selected, all to the disadvantage of Blacks [21]. In a study on a national sample of Black youth, in African American males, high household income was associated with higher risk of lifetime, 12-month, and 30-day major depressive disorder (MDD). The same risk associated with high SES was absent for African American females, and Caribbean Black males and females [20]. In another study among adults, high income was associated with higher risk of MDD in African American men [22]. In a nationally representative sample of Black adults, high education was associated with high suicidal ideation among Caribbean Black females but not Caribbean Black males or African American males or females [19].

One proposed mechanism as a potential explanation for the diminished health gain of Blacks from SES is discrimination [23]. In this view, high SES increases perception of discrimination at least in some sub-groups of Blacks. In a study using National Survey of American Life (NSAL)—Adults data, Hudson et al. found a positive interaction between education level and experiences of discrimination on depression, suggesting that experience of racial discrimination diminishes the effects of increased SES among African American men [24]. They have also shown high SES as a risk factor for MDD in NSAL data [25]. Similar findings could be replicated in the Coronary Artery Risk Development in Young Adults (CARDIA) study [26]. Fuller-Rowell has also suggested that the SES gain may be associated with some extra social, psychological, and physiological costs for Black youth [27,28].

To better understand the role of high SES as a vulnerability factor in the lives of minority youth, this study used a national sample of Black youth to investigate the effects of SES indicators on perceived discrimination based on ethnicity and gender intersection.

## 2. Methods

### 2.1. Design

Data of the National Survey of American Life—Adolescents (NSAL-A) supplement study was used [29,30,31]. Funded by the National Institute of Mental Health (NIMH), NSAL was conducted a part of the Collaborative Psychiatric Epidemiology Surveys (CPES).

### 2.2. Ethics

The NSAL study protocol was approved by the University of Michigan (UM) Institute Review Board (IRB). Assent was received from all the adolescents. Adolescents’ legal guardians also provided informed consent. Each respondent received $50 as financial compensation.

### 2.3. Participants and Sampling

The NSAL—Adolescent sample was drawn from the NSAL, a national probability sample of adult Blacks in the United States. The NSAL—Adult sample was screened for African American and Caribbean Black households with eligible adolescents living in the households. Adolescents living in households were randomly selected for participation. If more than one eligible adolescent lived in a household, two adolescents were selected based on the gender of the first eligible adolescent. As a result, adolescent data in NSAL are non-independent. The adolescent supplement data were weighted to adjust for non-independence of the selection probabilities and non-response at the household and individual levels. At the last step, the weighted data were post-stratified so the data can represent national estimates based on age, gender, and ethnicity [32,33].

### 2.4. Interview

All interviews were conducted in English language. Of all the interviews, 82% were face-to-face; the 18% remaining were conducted by telephone. Computer-assisted personal interviews (CAPI) were used in the face-to-face interviews. In CAPI, respondents use a computer to answer the questions. It is believed that CAPI improves data quality when a questionnaire is long and complex [34]. Each interview lasted 100 min on average. The response rate of the NSAL-A was above 80%.

### 2.5. Measures

The study measured ethnicity, age, gender, SES (subjective SES, income, and income to needs ratio), and perceived daily discrimination.

*Ethnicity.* NSAL-A measured family ethnicity as the self-identified ethnicity of the family household in which the adolescent lived. Participants self-identified as either African Americans or Caribbean Blacks. African American are defined as Black without having ancestral ties to the Caribbean. Caribbean Black was defined as Blacks having ancestral ties to a country included on a list of Caribbean countries provided by the interviewer or that their parents or grandparents were born in a Caribbean country. Caribbean countries included Antigua and Barbuda, Barbados, Bahamas, Cuba, Dominican Republic, Dominica, Grenada, Haiti, Jamaica, Saint Vincent and the Grenadines, Trinidad and Tobago, Saint Lucia, and Saint Kitts and Nevis.

*SES.* SES was measured using three indictors: financial hardship, family income, and income to needs ratio. To measure financial hardship, we asked participants if they have less than enough, enough, or more than enough money to live. We treated this variable as a dichotomous variable (less than enough versus other categories). Family income was measured using self-reported data, via interview by the parents. Income to needs ratio was measured in 6 levels based on dividing family income to number of individuals in the household. Higher income and income to needs ratio reflected higher SES, however, a value of 1 for financial hardship reflected low SES (0 for no financial hardship).

*Perceived Discrimination.* Perceived everyday discrimination was measured in the NSAL-A using a 13-item modified version of the Everyday Discrimination Scale (EDS). These items assess chronic, routine, and less overt discriminatory experiences that have occurred over the past year [35]. Although the original measure includes ten items, NSAL-A has added three additional items that reflect perceived teacher discrimination. Although this measure was originally developed and normed among adults, it also operates well for adolescents [35,36,37,38]. Respondents were asked: “In your day-to-day, life how often have any of the following things happened to you?” Sample items include: “being followed around in stores,” “people acting as if they think you are dishonest,” “receiving poorer service than other people at restaurants,” and “being called names or insulted.” The Likert response scale ranged from 1 (never) to 6 (almost everyday). A sum score was calculated, reflecting the frequency of exposure to discriminatory events over the past year (α = 0.86).

### 2.6. Statistical Analysis

To accommodate the NASL-A complex sampling design, we used Stata 13.0 (Stata Corp., College Station, TX, USA) to analyze the data. Taylor series approximation was used to recalculate the complex design-based estimates of variance and Standard errors. Thus, all inferences that are reported here, and also all the percentages and the means reflect the NSAL-A’s complex design. All the percentages represent proportions to the nation. Adjusted regression coefficients (B), their associated 95% confidence interval (CI) and associated *p*-value levels were reported. All the *p*-values between 0.05 and 0.1 were considered marginally significant. *p*-Values less that 0.05 was considered as marginal significant [39].

Several survey linear regressions were used for multivariable analysis. Due to correlations between various SES indicators, we ran separate models for the effect of each SES indicator on discrimination. The three SES indicators were financial hardship, family income, and income to need ratio (poverty index). In all our models, the main independent variable was one SES indicator and main outcome was perceived (daily) discrimination. For the model in the pooled sample, we entered age, gender, and ethnicity as covariates. For stratified models, we used age as the covariate. Intersections of ethnicity and gender were considered as strata. First, we ran linear regressions in the pooled sample. Then we ran models across ethnic by gender groups.

## 3. Results

Table 1 describes age, SES (centered family income, financial hardship, and income to need ratio (poverty index)), and perceived discrimination in the pooled sample, as well as across ethnic by gender groups. Highest level of financial hardship was reported by Caribbean Black females. Highest level of discrimination was reported by Caribbean Black males.

Table 2 summarizes five linear regressions with perceived discrimination as the outcome, family income as the independent variable, and age as the covariate. The first model was estimated in the pooled sample. Four other models were conducted in each ethnicity by gender groups. In the pooled sample, family income was not associated with higher perceived discrimination. In ethnic by gender groups, higher level of family income was associated with higher perceived discrimination for Caribbean Black females. Family income was not associated with perceived discrimination for Caribbean Black males, and African American males and females (Table 2).

Table 3 summarizes five linear regressions with perceived discrimination as the outcome, income to needs ratio as the independent variable, and age as the covariate. The first model was estimated in the pooled sample. Four other models were conducted in each ethnic by gender groups. In the pooled sample, income to needs ratio was not associated with perceived discrimination. Based on our ethnic by gender groups, however, high income to need ratio was associated with high perceived discrimination for Caribbean Black females and African American males. Income to need ratio was not associated with perceived discrimination for Caribbean Black males or African American females (Table 3).

Table 4 summarizes five linear regressions with perceived discrimination as the outcome, financial hardship as the predictor, and age as the covariate. The first model was estimated in the pooled sample. Four other models were conducted in each ethnicity by gender groups. In the pooled sample, financial hardship was marginally associated with higher perceived discrimination. In ethnic by gender groups, financial hardship was marginally associated with higher perceived discrimination for African American males and African American females but not Caribbean Black males and Caribbean Black females (Table 4).

## 4. Discussion

Using a national sample, the current study explored ethnic by gender differences in the pattern of the association between SES indicators and perceived discrimination of Black youth. At least two major results are found. First, patterns of the associations between SES and perceived discrimination across gender by ethnic groups of Blacks, as well as across SES indicators. Second, for African American males and Caribbean Black females, high SES may be a vulnerability factor that increases perceived discrimination.

This is not the first report on positive association between SES and perceived discrimination among Blacks. It is, however, the first to document ethnic by gender heterogeneities in these effects among Black youth. A recent study suggested that high subjective SES may be a vulnerability factor in African America youth, meaning a stronger association between discrimination and MDD in those with high subjective SES [40]. These findings help us understand why high SES is associated with worse mental health outcomes in some groups of Blacks [18,40]. For instance, among African American men, Hudson and colleagues have found a positive interaction between education level and experiences of major discrimination on depression. Their results suggest that experiencing high levels of racial discrimination diminishes the protective effects of high SES among African American men [24]. There are, however, very few studies showing high SES as vulnerability factor in Black youth and we are not aware of any study on this phenomenon among Caribbean Black females.

In a recent study, high family income was associated with higher risk of MDD among male but not female African American youth. This finding was consistent for lifetime, 12-month, and 30-day MDD [21]. In another study, high family income and living in predominantly White areas were associated with higher levels of discrimination among Black youth over a long period of time [41]. High SES as a vulnerability factor seems not to be limited to youth, as it is also found in other age groups [22]. It is also not specific to the effect of discrimination on MDD, as it extends to a wide range of health outcomes [18,19,21]. Another recent study showed that the effect of discrimination on poor mental health is larger for Blacks compared to other racial and ethnic minority groups in the U.S. [42].

We found that Caribbean Black females from higher income families report higher discrimination than Caribbean Black females from lower income families. For African American males and females, financial hardship and discrimination showed marginally significant association. Variations in the social contexts and history of different sociodemographic sub-groups of Blacks in the U.S. result in variation in the effects of social determinants on their health and well-being [43,44,45,46]. Thus, their life conditions are not merely the result of their racial category but a wide range of other factors such as ethnicity, culture, SES, and values [47]. Life conditions of Caribbean Blacks and African Americans are vastly different in the United States. The within-race heterogeneities reported here suggest that researchers, practitioners, and policy makers should be very cautious and not aggregate all American Blacks into a single racial category and assume that there are no within group heterogeneities in this population.

Higher discrimination in high SES Black youth may explain the results by Fuller-Rowell and colleagues who found a weaker health effect of educational attainment for African American than for White youth [28]. These findings also explain the Blacks’ diminished health gain and higher psychological costs of upward social mobility in Blacks [48]. In another longitudinal study using ACL data, high education credentials at baseline was a risk factor for an increase in depressive symptoms over the 25-year follow up period among African American men, an effect which could not be found in any other groups. Among African American males, higher years of schooling were still protective against an increase in depressive symptoms [18]. Literature has also shown that John Henryism, a coping style commonly used by Blacks for upward social mobility, may come with psychological costs [49,50,51]. Interestingly, similar to this study that showed high SES is a vulnerability factor for Caribbean Black females, previous studies have shown that high SES is associated with high suicidal ideation in Caribbean Black females but not other groups [19] and education increases risk of future depressive symptoms for African American males but not other groups [18].

### 4.1. Directions for Future Research

Future research should test whether discrimination mediates the effects of high SES on poor mental health of Blacks. There is also a need for future research to uncover mediators that explain ethnic variations in the link between SES and perceived discrimination. Culture, values, social norms, attribution style, racial and ethnic identity, and coping may have a role in differences in vulnerability of African American and Caribbean youth to discrimination as their SES changes. Future research could test the role of family types, race socialization, and other contextual factors such as density of Blacks in the neighborhoods in shaping the heterogeneities observed in this study.

### 4.2. Theoretical Implications

These findings also contribute to the existing theoretical knowledge regarding the role of SES in health disparities. Most existing theories such as Fundamental Cause Theory (FCT), focus on the health gain (not psychological costs) that follows high SES. These models have traditionally conceptualized high SES as protective factors [11,12,13,14]. We argue that whether SES operates as a risk or protective factor depends on population, context, social structure, SES indicator, and outcome. At least in some cases, there may be hidden risks associated with high SES for Blacks. Thus, there are instances that high SES comes with an extra cost of discrimination for Black youth.

### 4.3. Limitations

Our findings should be interpreted with a full consideration of the study limitations. First, we only controlled for a few confounders, and important variables such as parental education, socialization, family structure, living place, and contextual factors were not included. Second, our study was cross-sectional in design, thus any causative inference should be avoided [52]. There is a need to replicate these findings using other independent datasets, settings, and age groups [53,54,55,56]. Despite these limitations, the findings reported here contribute to the literature, as very few studies have conceptualized high SES as a vulnerability factor among minorities.

## 5. Conclusions

Our findings suggest that at least for some sub-groups of Black youth, high SES may be a vulnerability factor, as high SES may increase exposure to discrimination. The effects are, however, specific to gender by ethnic groups, and also the SES indicators. As a result, how discrimination contributes to poor mental health of Black youth is complex and depends on ethnicity, gender, and SES. This suggests that the underlying mechanisms for health disparities are complex and the effects are non-linear [57]. Future research should investigate why particular SES indicators in particular groups increase perceived discrimination. Future research should also examine whether these effects are due to differential exposure to discrimination, or a change in attribution of ambiguous exposures to racial situations.

## Figures and Tables

**Table 1:** Descriptive statistics.

	All		African American Female		African American Male		Caribbean Black Female		Caribbean Black Male	
	Mean	95% CI	Mean	95% CI	Mean	95% CI	Mean	95% CI	Mean	95% CI
Age (Years)	14.97	14.84–15.09	14.91	14.72–15.10	14.99	14.83–15.15	15.55	15.44–15.66	14.80	14.59–15.01
Family Income (Centered)	170.31	−4159.66–4500.27	196.85	−4874.99–5268.70	83.65	−6101.89–6269.19	−478.97	−8941.67–7983.74	1930.03	−7151.01–11,011.08
Income to Needs Ratio	3.98	3.73–4.23	3.98	3.74–4.21	3.95	3.58–4.33	3.99	3.614.38	4.43	3.58–5.27
Financial Hardship										
No	86.89	83.68–89.55	89.21	85.11–92.28	87.05	78.56–92.50	69.95	52.71–82.93	85.63	80.55–89.55
Yes	13.11	10.45–16.32	10.79	7.72–14.89	12.95	7.50–21.44	30.05	17.07–47.29	14.37	10.45–19.45
Perceived Discrimination (Everyday)	5.07	4.68–5.47	4.76	4.31–5.21	5.36	4.81–5.91	4.48	3.75–5.22	6.13	4.25–8.01

Confidence Interval (CI).

**Table 2:** Summary of linear regression on the effects of family income on perceived discrimination.

	All		African American Female		African American Male		Caribbean Black Female		Caribbean Black Male	
	B	95% CI	B	95% CI	B	95% CI	B	95% CI	B	95% CI
**Ethnicity (Caribbean Black)**	−0.01 ^#^	−0.03–0.00	-	-	-	-	-	-	-	-
**Gender (Female)**	0.00	0.00–0.01	-	-	-	-	-	-	-	-
**Age (Years)**	0.01 *	0.00–0.02	0.12	−0.23–0.47	0.59 ***	0.36–0.82	0.25	−0.09–0.60	−0.05	−0.66–0.55
**SES (Family Income)**	0.00	0.00–0.00	0.00	0.00–0.01	0.00	0.00–0.01	0.01 *	0.00–0.03	0.00	−0.03–0.04
**Intercept**	−0.05	−0.14–0.04	2.97	−2.34–8.29	−3.50 ***	−6.88–−0.12	0.60	−5.14–6.34	6.86	−3.66–17.38

Outcome: Discrimination (Everyday), Socioeconomic Status (SES), Confidence Interval (CI); ^#^ *p* < 0.1, * *p* < 0.05, *** *p* < 0.001.

**Table 3:** Summary of linear regression on the effects of income to needs ratio on perceived discrimination.

	All		African American Female		African American Male		Caribbean Black Female		Caribbean Black Male	
	B	95% CI	B	95% CI	B	95% CI	B	95% CI	B	95% CI
**Ethnicity (Caribbean Black)**	−0.01 *	−0.03–0.00	-	-	-	-	-	-	-	-
**Gender (Female)**	0.00	0.00–0.01	-	-	-	-	-	-	-	-
**Age (Years)**	0.01 ^#^	0.00–0.02	0.13	−0.23–0.48	0.61 ***	0.38–0.83	0.27	−0.13–0.66	−0.09	−0.66–0.48
**SES (Income to needs ratio)**	0.00	−0.01–0.01	0.12	−0.06–0.31	0.20 *	0.02–0.37	0.35 ***	0.17–0.53	0.35	−0.25–0.95
**Intercept**	−0.06	−0.14–0.02	2.43	−2.91–7.76	−4.53 **	−7.82–−1.25	−1.11	−7.29–5.07	5.89	−2.17–13.96

Outcome: Discrimination (Everyday), Socioeconomic Status (SES), Confidence Interval (CI); ^#^ *p* < 0.1, * *p* < 0.05, ** *p* < 0.01, *** *p* < 0.001.

**Table 4:** Summary of linear regression on the effects of financial hardship on perceived discrimination.

	All		African American Female		African American Male		Caribbean Black Female		Caribbean Black Male	
	B	95% CI	B	95% CI	B	95% CI	B	95% CI	B	95% CI
**Ethnicity (Caribbean Black)**	−0.01 ^#^	−0.03–0.00	-	-	-	-	-	-	-	-
**Gender (Female)**	0.00	−0.01–0.00	-	-	-	-	-	-	-	-
**Age (Years)**	0.01 ^#^	0.00–0.01	0.11	−0.25–0.46	0.60 ***	0.36–0.83	0.30 ^#^	−0.03–0.63	−0.02	−0.50–0.45
**SES (Financial hardship)**	0.04 **	0.01–0.07	1.22 ^#^	−0.17–2.61	1.76 ^#^	−0.02–3.54	0.70	−0.66–2.05	1.80	−0.64–4.24
**Intercept**	−0.04	−0.13–0.05	3.09	−2.28–8.46	−3.69 *	−7.17–−0.21	−0.24	−5.49–5.02	5.93	−1.68–13.54

Outcome: Discrimination (Everyday), Socioeconomic Status (SES), Confidence Interval (CI); ^#^ *p* < 0.1, * *p* < 0.05, ** *p* < 0.01, *** *p* < 0.001.
